# Deletion of Running-Induced Hippocampal Neurogenesis by Irradiation Prevents Development of an Anxious Phenotype in Mice

**DOI:** 10.1371/journal.pone.0012769

**Published:** 2010-09-16

**Authors:** Johannes Fuss, Nada M. B. Ben Abdallah, Frank W. Hensley, Klaus-Josef Weber, Rainer Hellweg, Peter Gass

**Affiliations:** 1 Department of Psychiatry and Psychotherapy, Central Institute of Mental Health Mannheim (ZI), University of Heidelberg, Mannheim, Germany; 2 Department of Radiooncology, University of Heidelberg, Heidelberg, Germany; 3 Department of Psychiatry, University of Medicine of Berlin, Campus Charité Mitte, Berlin, Germany; UMR CNRS 5226 - Université Bordeaux 2, France

## Abstract

Recent evidence postulates a role of hippocampal neurogenesis in anxiety behavior. Here we report that elevated levels of neurogenesis elicit increased anxiety in rodents. Mice performing voluntary wheel running displayed both highly elevated levels of neurogenesis and increased anxiety in three different anxiety-like paradigms: the open field, elevated O-maze, and dark-light box. Reducing neurogenesis by focalized irradiation of the hippocampus abolished this exercise-induced increase of anxiety, suggesting a direct implication of hippocampal neurogenesis in this phenotype. On the other hand, irradiated mice explored less frequently the lit compartment of the dark-light box test irrespective of wheel running, suggesting that irradiation *per se* induced anxiety as well. Thus, our data suggest that intermediate levels of neurogenesis are related to the lowest levels of anxiety. Moreover, using c-Fos immunocytochemistry as cellular activity marker, we observed significantly different induction patterns between runners and sedentary controls when exposed to a strong anxiogenic stimulus. Again, this effect was altered by irradiation. In contrast, the well-known induction of brain-derived neurotrophic factor (BDNF) by voluntary exercise was not disrupted by focal irradiation, indicating that hippocampal BDNF levels were not correlated with anxiety under our experimental conditions. In summary, our data demonstrate to our knowledge for the first time that increased neurogenesis has a causative implication in the induction of anxiety.

## Introduction

The relation between anxiety and hippocampal activity has been subject to research for many years [1], [2]. Most of the investigations done so far were performed in rodents, since anxiety is an old emotional state in phylogenetic history observed across all mammalian species [3]. This is also reflected by the strong resemblance of the definitions of anxiety or *Angst* in different disciplines including biology, psychology, and philosophy [2], [4], [5], highlighting the common plinth. Anxiety is basically an emotion aroused by ambiguous or potential threats and situations which are not fully assessable. An anxious state is characterized by conflicting approach/avoid tendencies to unconditioned and diffuse adversity. Though anxiety manifestations are an essential ingredient of life, they can extend to different forms of psychopathologies.

The core brain structures implicated in the processing of anxiety are old in phylogenetic perspective and preserved in both human and murine brains [3]. The involvement of hippocampal function in anxiety has been demonstrated by lesioning and pharmacological studies, with a primary focus on the ventral part of the hippocampus [1], [2]. Neurogenesis occurs continuously in two neurogenic niches in adult mammals, either of them is the hippocampus [6], [7]. In the last years, the role of hippocampal neurogenesis in emotional behavior has been a matter of extensive debate [8]. While several findings proposed a key function in depressive behavior [9], recent evidence also indicates that the genesis of new cells in the subgranular zone of the dentate gyrus is part of the biological underpinnings mediating anxiety. Indeed, several gain and loss-of-function studies in adult rodents reported an effect of hippocampal neurogenesis on anxiety behavior [10], [11], [12], [13]. Recently, we reported a strong positive correlation of increased neurogenesis and elevated anxiety-levels in running mice [10], which was also corroborated in rats [14]. These findings challenged the view of a general protective function of elevated hippocampal neurogenesis in mood disorders.

The aim of the present study was to investigate whether the correlation of neurogenesis and anxiety that we previously described in running mice was just an epiphenomenon or represented a causative relation. While voluntary running increases hippocampal neurogenesis in rodents significantly [15], cranial irradiation restricted to the hippocampus results in strong and long-term reductions of adult neurogenesis, depending on the administered dose [16]. We combined both running and irradiation in mice to (i) investigate the impact of increased or decreased neurogenesis on anxiety-related behaviors and to (ii) explore whether the observed increase in anxiety in running mice is a direct consequence of increased hippocampal neurogenesis or is based on other running-induced brain alterations such as protein expression of the neurotrophin BDNF [10], [17].

## Results

### Previous irradiation decreases cell proliferation and neurogenesis per se and after voluntary running

**Figure 1:**
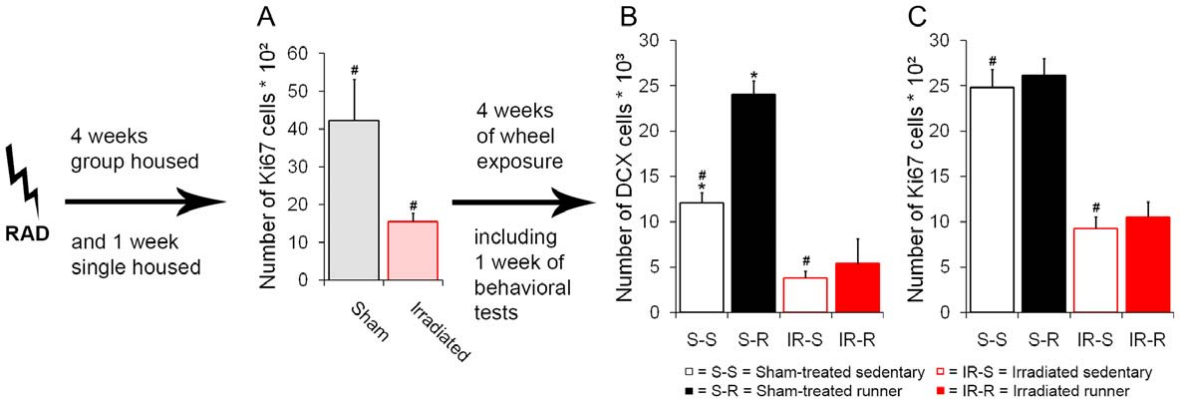
Experimental scheme and effects of focal hippocampal irradiation on cell proliferation and neurogenesis. (**A**) Radiation decreased cell proliferation significantly. This decrease persisted even after voluntary wheel running. (**B**) Running induced a significant increase in DCX positive cells in sham-treated runners, while both irradiated groups had significantly less DCX-cells within the dentate gyrus. (**C**) In contrast, running had no impact on the number of Ki-67-positive cells in sham-treated mice. *, *°*, *^#^ indicate significant post-hoc differences between sham-treated sedentary and runners*, *sham-treated runners and irradiated runners*, *sham-treated sedentary and irradiated mice*, *respectively*; *columns represent means + standard error*.

At the age of 4 weeks, half of the mice (n = 36) received a single hippocampus-focalized irradiation dose of 10 Gy. Four weeks after irradiation, hippocampal proliferation was assessed by Ki67 immunocytochemistry in a subgroup of 8 mice. The number of Ki67-positive cells in the subgranular zone was significantly reduced in irradiated animals (n = 4) compared to their sham-treated sedentary controls (n = 4; p<0.05; Fig. 1A + 2A). One month after irradiation, irradiated (n = 32) and sham-treated mice (n = 32) were single housed and, one week later, their cages were equipped with functioning (n = 32) or blocked (n = 32) running wheels. Running distances in both irradiated (IR-R; n = 16) and sham-treated (S-R; n = 16) runners were comparable (Mean total distance IR-R  = 8.4±0.4 km/day; S-R  = 7.8±0.4 km/day; p = 0.23) during 33 days of running. Furthermore, the circadian rhythm of running activity as previously described [10] was not affected by irradiation (Fig. 3).

**Figure 2 pone-0012769-g002:**
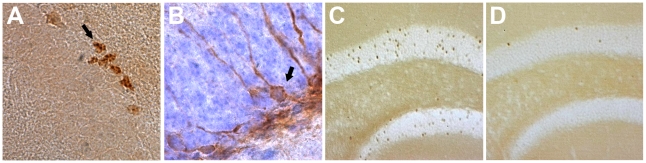
Histological stainings used in this study. Pictures illustrating the staining for neurogenesis (A =  Doublecortin), cell proliferation (B =  Ki67), and c-Fos activity in a running (C) and sedentary mouse (D). Black arrows point at representative cells for the counting procedure.

**Figure 3 pone-0012769-g003:**
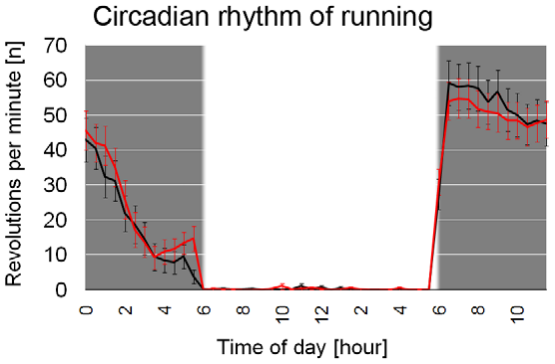
Hippocampal irradiation is not affecting the circadian rhythmicity of running. All runners were highly active during the dark phase of the light-dark-rhythm as noted by their running activity. Additionally, no running differences were observed between sham-treated (black line) and irradiated (red line) runners. The graph illustrates the running pattern of the last day of running (day 20) displaying averaged values in 30 min segments. Bars represent standard errors.

Nine weeks after irradiation (i.e., 4 weeks after the introduction of running wheels), cell proliferation and neurogenesis were measured in the dentate gyrus of all experimental groups (n = 6/group; Fig. 1). In line with our previous findings (11), four weeks of running had no significant influence on the number of cells expressing the nuclear proliferation marker Ki67 in the subgranular zone of the dentate gyrus (Fig. 1C), while doublecortin-positive cells (DCX) were significantly increased (F_1,20_ = 16.09; p = 0.001; Fig. 1B). The number of immature neurons expressing the filament protein doublecortin was significantly reduced in both irradiated groups (runners and non-runners), compared to sham-treated mice (F_1,20_ = 63.90; p<0.001; Fig. 1B). It is worth noting that 4 weeks of voluntary wheel running yielded no increase in neurogenesis in irradiated animals (p = 0.505; Fig. 1), demonstrating the long-lasting diminishing effect of irradiation on neurogenesis. In addition, the number of cells expressing the nuclear proliferation marker Ki67 was still reduced 9 weeks after irradiation in both runners and non-runners (F_1,33_ = 79.39; p<0.001; Fig. 1C). To exclude an effect of behavioral testing on proliferation and neurogenesis, 4 animals per group remained behaviorally naive until the day of sacrifice. There was no measurable effect of behavioral testing on the number or morphology of immunostained cells.

### Irradiation prevents running-induced anxiety

**Figure 4 pone-0012769-g004:**
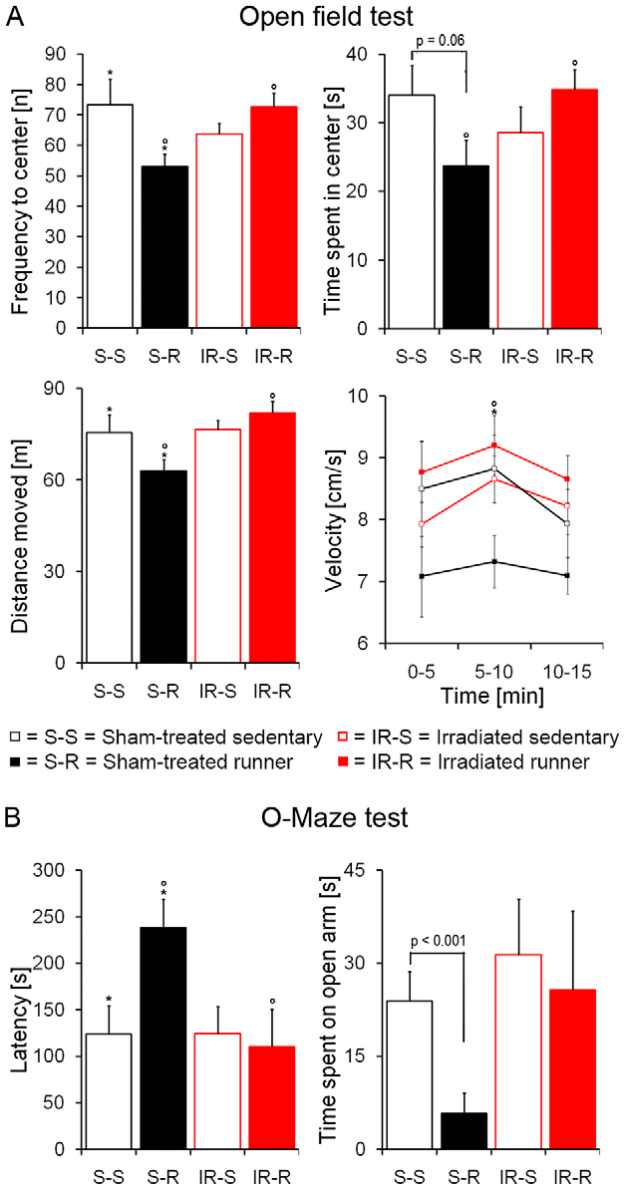
Voluntary running produces an anxious phenotype in sham-treated, but not in irradiated mice. (**A**) Sham-treated runners had significantly less approaches to the center of the open field and moved a lower total distance. Sham-treated runners spent significantly less time in the center of the open field and exhibited delayed habituation. (**B**) Latencies to venture out on the open arm of the O-maze were higher in sham-treated runners compared to sham-treated sedentary controls and to irradiated runners. Consistently, the total time spent on the open, unprotected arms was shorter in sham-treated runners compared to sham-treated sedentary controls, while both irradiated groups performed comparably. *, *°*, *^#^ indicate significant post-hoc differences between sham-treated sedentary and runners*, *sham-treated runners and irradiated runners*, *sham-treated sedentary and irradiated mice*, *respectively*; *columns represent means + standard error*.

The following aim was to define whether the irradiation-induced reduction of neurogenesis hinders the development of an anxious phenotype by voluntary wheel running. We have indeed recently reported a positive correlation between running-induced increase of neurogenesis and anxiety behavior [10]. Ablating hippocampal neurogenesis by irradiation should illuminate whether this correlation was just an epiphenomenon or a causal factor. First, mice were subjected to an open field test: In line with previous results, sham-treated runners, with high levels of neurogenesis, exhibited less activity and reduced exploratory behavior compared to sedentary controls (Fig. 4A). Irradiated runners, however, performed comparably to sham-treated and irradiated sedentary controls, all of which bearing lower levels of neurogenesis than sham-treated runners (Fig. 4A). Sham-treated runners had significantly less approaches to the center of the open field (F_1,44_ = 6.85; p = 0.01) and spent significantly less time therein during the first 5 min of the test (F_1,44_ = 4.90; p = 0.03; Fig. 4A) and over the total time course (F_1,44_ = 5.51; p = 0.02). Moreover, sham-treated runners traveled a significantly lower distance in the open field (F_1,44_ = 4.45; p = 0.04; Fig. 4A), notably this effect was not due to a generally reduced locomotion in running mice (see below). A time course analysis revealed furthermore a slower habituation of sham-treated runners when comparing the animals' velocity. Between 0–5 min only a slight difference emerged in sham-treated runners (F_1,44_ = 3.61; p = 0.06). This trend became more pronounced in the second time bin between 5–10 min (F_1,44_ = 4.96; p = 0.03; Fig. 4A), while it was absent between 5–15 min indicating a delayed habituation of sham-treated runners (F_1,44_ = 2.17; p = 0.15). With these data we thus confirmed earlier described behavioral changes induced by voluntary running in untreated animals in the open field test [10]. We further show that these running-linked effects were completely abolished by irradiation.

In a second step, approach-avoidance conflicts were measured in the elevated O-maze, an established test for generalized anxiety. Mice were placed on a sheltered sector of the maze, and time and frequency of venturing on anxiety-related open sectors were recorded. The latencies to approach the aversive sectors of the O-maze were increased in sham-treated runners - confirming previous findings [10] - but not in irradiated runners (F_1,44_ = 3.73; p = 0.06; Fig 4B). Two-factorial ANOVA revealed no significant interaction between irradiation and running when analyzing the total time spent on the aversive open sector. Since we observed a significant difference between runners and controls before [10], we again performed Student t-test and observed a significant difference between both sham-treated groups (p<0.001). This difference was absent in irradiated mice (p = 0.72; Fig. 4B).

**Figure 5:**
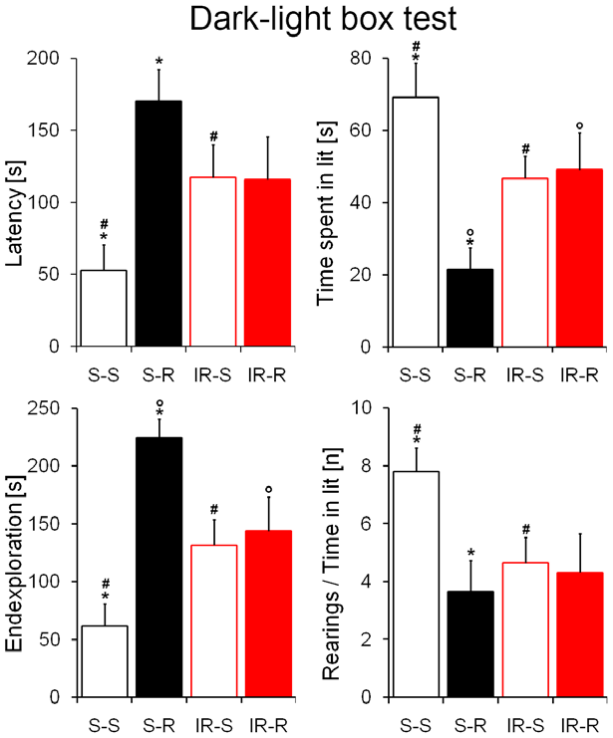
Both running and irradiation increase anxiety in the dark-light box. Sham-treated runners exhibited an anxious phenotype when compared to sham-treated sedentary controls. Furthermore, irradiated mice were significantly more anxious than sham-treated sedentary controls in all parameters investigated. Nevertheless, irradiated runners exhibited less anxiety than sham-treated runners (time spent in lit p<0.05; endexploration time p<0.05). *, *°*, *^#^ indicate significant post-hoc differences between sham-treated sedentary and runners*, *sham-treated runners and irradiated runners*, *sham-treated sedentary and irradiated mice*, *respectively*; *columns represent means + standard error*.

Mice were subsequently tested in the dark-light box, another paradigm of approach-avoidance behavior. Sham-treated runners displayed reduced exploration of the aversive light compartment with higher latencies to visit (F_1,44_ = 6.47; p = 0.01; Fig. 5) and less time spent in the bright compartment (F_1,44_ = 9.32; p = 0.004; Fig. 5). Furthermore the time to approach the end of the lit chamber was significantly altered in sham-treated runners (F_1,44_ = 11.43; p = 0.002; Fig. 5). The number of rearings divided through the time in the brightness was comparable between irradiated mice and sham-treated runners, thus ANOVA revealed an influence of running on performance (F_1,44_ = 4.54; p = 0.04; Fig. 5) and a non significant interaction effect (F_1,44_ = 3.27; p = 0.08). Interestingly, unlike the previous paradigms, where irradiated mice performed comparably to sham-treat sedentary controls, post-hoc analyses revealed differences between sham-treated sedentary controls and both groups of irradiated mice in the dark-light box, with irradiated mice exhibiting increased anxiety compared to sedentary controls (Latency p = 0.03; Time spent in lit p = 0.04; Endexploration time p = 0.008; Rearings/time in lit p = 0.01; Fig. 5).

### Irradiation mitigates the running-related downregulation of c-Fos expression during anxiety

**Figure 6:**
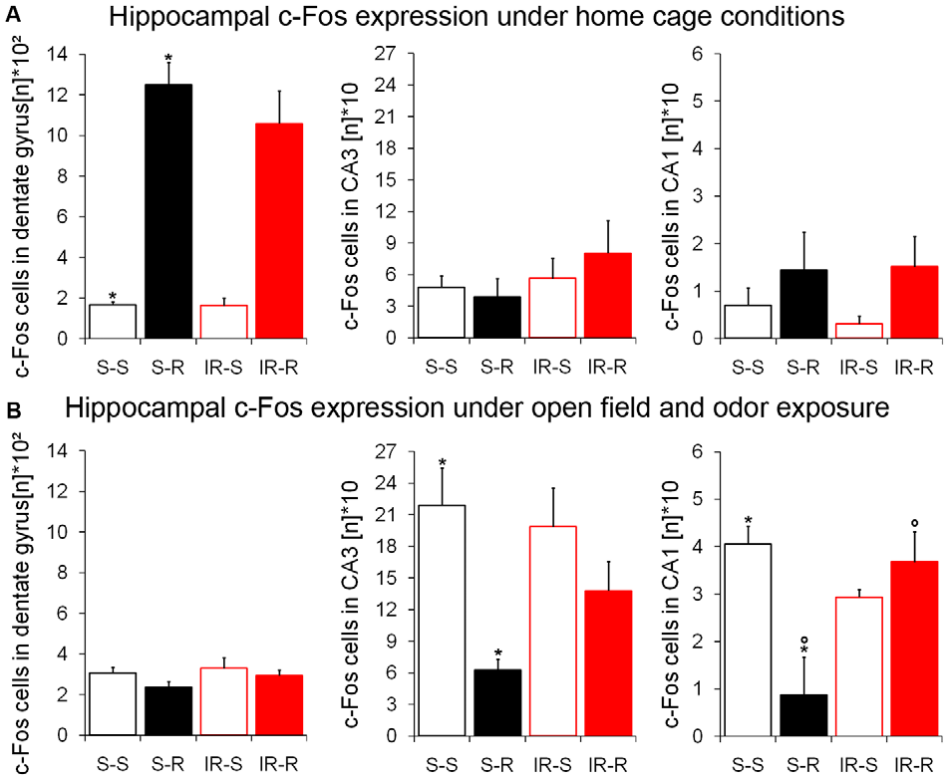
When exposed to an anxiogenic situation, sham-treated runners have a different c-Fos expression in the hippocampus. (**A**) In control mice not exposed to the cat odor open field, c-Fos activation was remarkably higher in the dentate gyrus and CA1 region of regularly running mice, irrespective of irradiation. (**B**) After exposure to cat odor, runners had reduced numbers of c-Fos positive cells in CA3. This effect of running was completely abolished by previous irradiation in CA1. *, *°*, *^#^ indicate significant post-hoc differences between sham-treated sedentary and runners*, *sham-treated runners and irradiated runners*, *sham-treated sedentary and irradiated mice*, *respectively*; *columns represent means + standard error*.

Potential threats, e.g. when mice must enter an area where a cat has been or might be, represented by its odor, elicit anxiety behavior and hippocampal activation [18]. Response to such situations is affected by anxiolytic drugs which act mainly on the activity of the septo-hippocampal system [2], [18]. In order to examine hippocampal activity in such an aversive environment, 24 mice (n = 6 from each group) were exposed to a brightly lit open field with a tube emitting cat odor from fecal samples placed in the center of the arena. To validate the specificity of c-Fos expression to the exposure, we used cage controls that were not exposed to the “open field + odor” arena, but anesthetized and perfused at the same time and day. Running wheels were freely available for control mice, since runners were performing more than 95% of exercise during the dark phase of the light-dark cycle. Experiments were done in the middle of the light phase. These cage control animals showed a strong effect of regular running on c-Fos expression in the hippocampus (Fig. 6A). This effect was most pronounced in the “input” element of the trisynaptic excitatory circuit (dentate gyrus -> CA3 -> CA1), namely dentate gyrus with more than 6 fold increase in c-Fos positive cells in both sham-treated and irradiated runners compared to both sedentary groups (F_1,12_ = 84.90; p<0.001; Fig. 6A). No significant difference was detectable in CA3. Running had furthermore a less pronounced impact on c-Fos expression in the output subregion of ventral CA1 (F_1,12_ = 3.85; p = 0.073; Fig. 6A).

Exposure to the aversive context strongly increased neuronal activation as shown in c-Fos protein expression in sham-treated sedentary controls, confirming the anxiogenic nature of the odor stimulus (Fig. 6B). Interestingly, c-Fos expression in the dentate gyrus was no longer affected by previous running after anxiety exposure, (F_1,17_ = 1.99; p = 0.176). In contrast, two-factorial ANOVA revealed a significant reduction of c-Fos positive cells in CA3 of runners after anxiety exposure (F_1,17_ = 11.36; p = 0.004; Fig. 6B). Interestingly, this effect was most pronounced in sham-treated runners, while the number of cells in irradiated runners was on an intermediate level between sham-treated runners and both groups of sedentary controls. Nevertheless, post-hoc comparison indicated no significant difference between both group of runners (p = 0.141). In contrast, odor exposure significantly decreased c-Fos expression in CA1 only in sham-irradiated runners. Thus we found an interaction of irradiation and running on cell numbers in CA1 (F_1,17_ = 5.12; p = 0.037; Fig. 6B). Post-hoc comparison revealed higher numbers of cells in irradiated runners (p<0.05) and sham-treated sedentary controls (p<0.05) compared to sham-treated runners. The expression of c-Fos in irradiated runners was statistically indistinguishable from irradiated and sham-treated sedentary controls. Thus, hippocampal irradiation blocked the running-related downregulation of c-Fos expression in a situation of strong anxiety in CA1 but not CA3.

### Running-induced BDNF elevation does not correlate with anxiety levels

**Figure 7:**
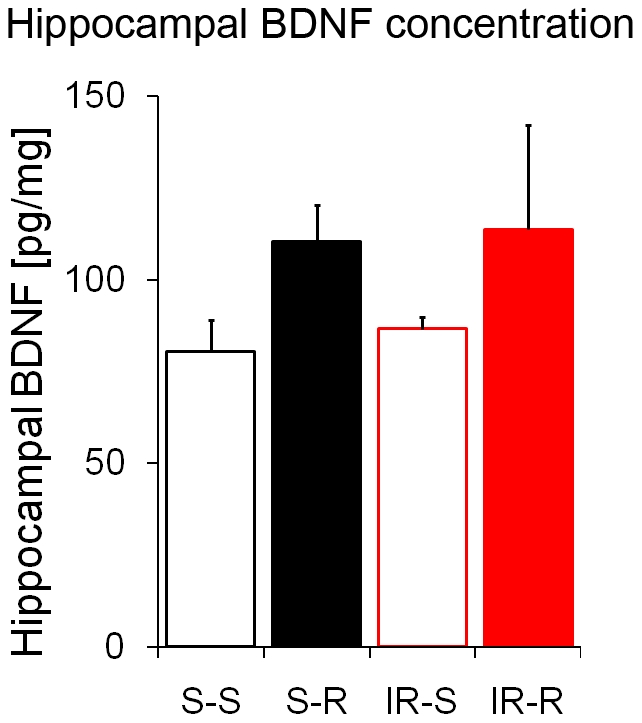
Hippocampal BDNF increases in runners irrespective of irradiation. Four weeks of voluntary running increased hippocampal brain-derived neurotrophic factor (BDNF) levels significantly. Though irradiation affected neurogenesis dramatically, it had no impact on the concentration of BDNF. *Columns represent means + standard error*.

As demonstrated, irradiation of the hippocampus was sufficient to abolish the effects of running on neurogenesis and anxiety. Besides neurogenesis, BDNF has been hypothesized to have a crucial effect on anxiety [10], [19], [20], [21]. We therefore measured hippocampal BDNF levels in all four experimental groups. As expected by previous studies [10], [17] BDNF increased in the hippocampus after long-term voluntary exercise (F_1,20_ = 4.91; p = 0.04; Fig. 7). In contrast to its effect on neurogenesis and anxiety-related behaviors, irradiation did not affect the levels of BDNF in the hippocampus, neither in running nor in sedentary animals (F_1,20_ = 0.14; p = 0.72).

### Running and sedentary mice exhibit comparable home cage activity

**Figure 8:**
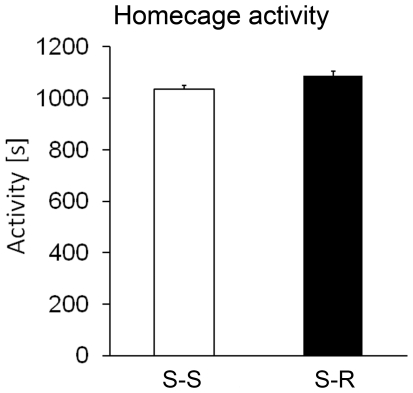
Locomotor home cage activity is not altered by voluntary running. After 21 days of running, both running and sedentary mice exhibited comparable home cage activity. Thus running mice do not display hypo-locomotion under familiar conditions, ruling out a confounding factor for anxiety testing. *Columns represent means + standard error*.

Sham-treated runners were less active than all other groups in the open field, O-maze and dark-light test. To exclude a general locomotor deficit induced by voluntary running, we investigated the basal home cage activity in a separate group of runners (n = 12) and sedentary controls (n = 12) after 21 days of running. Both groups exhibited the same locomotor activity during 8 hours of testing (Fig. 8). A time course analysis revealed furthermore no significant alteration in relation to the time of testing.

## Discussion

Our results validate the anxiogenic effect of elevated neurogenesis after voluntary running. Here we were able to show that running-induced anxiety does not occur after hippocampus-focalized irradiation, demonstrating the essential contribution of neurogenesis. In contrast, hippocampal BDNF levels were not altered by irradiation and thus did not correlate with the development of an anxious phenotype. Interestingly, we observed a difference in hippocampal c-Fos activation in sham-treated runners following exposure to an environment soliciting anxiety, suggesting an additional functional correlate of the increased anxiety. Together these findings are in accordance with the concept that the hippocampus is a core structure in the genesis of anxiety [1], [2]. It is indeed an accepted notion that decreased hippocampal activity following lesions or applications of various anxiolytics results in decreased anxiety behavior [1], [2], [22]. However, it has been less investigated whether elevated hippocampal activity coincides with increased anxiety.

The functional incorporation of adult-born neurons into hippocampal circuits increases hippocampal activity [23]. Animals with higher hippocampal neurogenesis perform better in tests that are highly sensitive for hippocampal function in learning and memory [15], [24], while decreased hippocampal neurogenesis results in poorer performances [25], [26], [27], [28], albeit a recent report challenges this view [29]. Together, these findings indicate a strong relation of hippocampal neurogenesis with increased hippocampal activity and cognitive performance.

The connection between anxiety and increased neurogenesis as a modulator of hippocampal functioning is less clear. In an earlier report we found a positive correlation for hippocampal neurogenesis and anxiety in voluntary runners [10]. In the present study we corroborated this finding and validated it by blocking hippocampal neurogenesis. Sham-treated runners showed increased anxiety after long-term voluntary running in 3 different ethologically-based tests. In contrast, irradiated runners exhibited similar anxiety behavior compared to irradiated sedentary mice. Thus we conclude that increased neurogenesis is a crucial mechanism by which anxiety is induced in voluntarily running mice. The anxiogenic effect of voluntary running was also described in rats recently [14]. The finding that improved cognition and high trait anxiety both occur in rodents with high neurogenesis might be counterintuitive at a first glance. But it should be borne in mind that anxiety is a highly cognitive state and not in general a detrimental emotion [4]. Anxiety has a profound evolutionary significance for the survival of individuals. Therefore increased anxiety should not be generally interpreted as a model for psychiatric pathology, e.g. wild mice display increased anxiety behavior in response to open areas compared to inbred laboratory strains [30]. Wild wood mice have moreover much higher neurogenesis rates in the dentate gyrus [31].

Previous reports demonstrated that ablation as well as increase of hippocampal neurogenesis may result in increased anxiety [11], [12], [13], suggesting that intermediate levels of neurogenesis are related to low trait anxiety. In our present study, we observed such a *U*-shaped relation for neurogenesis and anxiety in the dark-light box test. Irradiated mice showed increased anxiety and reduced neurogenesis compared to sham-treated sedentary controls, while sham-treated runners exhibited increased anxiety along with increased neurogenesis compared to sedentary controls. In contrast, neither the open field nor the O-maze test revealed an anxiogenic effect of irradiation. Of note, however, irradiation did not ablate neurogenesis completely in our experimental setting, thus the decrease in neurogenesis was milder compared to genetic approaches [12]. Consistently, in *Klf*-9-null mice decreased differentiation and plasticity of adult born neurons elicit anxiety which is also only detectable in a dark-light-paradigm and not in other anxiety-related tests [32]. These data suggest that the dark-light-paradigm may be more sensitive to deficits in hippocampal neurogenesis compared to other anxiety-related tests. Thus, our data show that physiological levels of neurogenesis may be important for anxiety processing and deviations from this range can result in increased anxiety behavior. Nevertheless, in some previous reports neither ablation nor increase of neurogenesis did affect anxiety-related behavior [9], [33]. In line with these reports, in two anxiety tests (open field and O-maze) irradiated mice performed comparably to sham-treated sedentary controls. Profound differences in neurogenesis in irradiated mice compared to sham-irradiated sedentary controls had therefore no influence on anxiety behavior in these tests. Irradiation did moreover not alter the running activity of mice during 4 weeks of voluntary wheel running. This is of particular importance, since general locomotor activity differences between mice could explain alterations in anxiety tests. To rule out a general hypo-locomotion in running mice, we performed a home cage activity measurement in another cohort of mice. These measurements revealed no significant difference between sedentary and running mice (Fig. 8), thus neither wheel running nor irradiation affected basal locomotion.

While irradiation resulted in reductions in the number of proliferating cells, we observed no influence of long-term voluntary running on the number of *Ki67*-positive cells, confirming thus our recent observation [10]. Though previous studies have reported a stimulating effect of running on proliferation under comparable conditions [34], our data are in line with a recent study reporting that proliferation is only increased at the onset of wheel running and returns to baseline levels after 21 days of running [35].

To investigate the influence of running and irradiation on hippocampal activation we further performed c-Fos staining of the hippocampal subregions. The dentate gyrus receives its main excitatory input from the perforant pathway. Different inputs mediating anxiety converge upon the neurons of the dentate gyrus, which in turn project with their axons to the CA3 region [36]. Adult born neurons in the dentate gyrus form synapses with hilar and CA3 neurons around the age of 2 weeks [37] influencing the activity of the input regions of the trisynaptic circuit. Following the hypothesis that altered hippocampal activity affects anxiety, we were aiming to find a functional cellular correlate in a situation of anxiety. Expression of the c-Fos gene is a robust correlate for neuronal activity [38]. Previous studies reported lower numbers of anxiety-induced c-Fos positive cells in rodents with higher trait anxiety [39], [40]. Consistently, we found less c-Fos cells in ventral CA1 following an anxiogenic stimulus in sham-treated runners (Fig. 6B), which exhibit both more anxiety and neurogenesis (Fig. 1–5). Moreover sham-treated runners failed to alter ventral CA1 activity when exposed to anxiety, while all other groups increased c-Fos expression. It is noteworthy, that the ventral CA1 region of Ammon's horn is the main output of the trisynaptic circuit to regions crucial for anxiety and neurogenesis processing [41], [42], [43]. At a first glance, these results seem to be at odd with the hypothesis of increased hippocampal activity due to increased neurogenesis in sham-treated runners. But one has to keep in mind that c-Fos expression does not give insight into the quality of activity, namely whether cells are excitatory or inhibitory. Approximately 70% of the target cells of mossy fibers of adult born neurons are inhibitory interneurons [44]. Mossy fibers have contacts with interneurons *en passant* before they reach the pyramidal cells of CA3. Acsády and colleagues (1998) averaged that granule cells in general have about 50 times more connections to inhibitory cells. Such anatomical connections might account for the reason why increased activity of granule cells is generally associated with reduction of pyramidal cell activity in CA3 [45], highlighting the intricate orchestration of the hippocampal circuitry.

Besides our findings of c-Fos expression under anxiety-inducing conditions, we observed a strong effect of regular voluntary running on dentate gyrus c-Fos activity in cage controls. Animals equipped with a wheel had 6 times more c-Fos cells in the dentate gyrus than sedentary controls, irrespective of irradiation. High dentate gyrus activity might be one underpinning of the increased neurogenesis in this neurogenic niche. Particularly, since cell activity also regulates ambient neurotransmitter levels which give first input to newborn neurons before synaptic innervations [46]. It is tempting to speculate that the continuous hyperactivation along with increased neurogenesis could have negative effects on the functioning of the hippocampal network. A resulting reduction in excitatory drive of neurons in the hippocampal subfield could be one compensational mechanism to adjust this activity gain. In this context, reduced c-Fos levels in animals with higher trait anxiety could be a consequence of synaptic scaling [47].

In conclusion, our data demonstrate that an excessive increase of neurogenesis has potential negative consequences as indicated in previous reports [29]. These data thus challenge the simplistic view that more newborn neurons are always better for mental health. However, many brain changes might have occurred following exercise and irradiation which affected neurogenesis in an indirect fashion leading to anxiety. In previous findings hippocampal BDNF levels were correlated with anxiety [10], [19], [20]. By blocking neurogenesis and thereby altering anxiety without any changes in BDNF levels, we could demonstrate that BDNF failed to correlate with anxiety. Further investigations are nevertheless still needed to determine the mechanism through which alterations in adult hippocampal neurogenesis induce anxiety.

## Materials and Methods

### Experimental animals

A total of 72 C57BL/6J male mice obtained at the age of 4 weeks from Charles River (Sulzfeld, Germany) were used to assess neurogenesis and anxiety after irradiation and running. Mice were irradiated upon their arrival in our animal facility and were kept for 4 weeks group-housed in a temperature and humidity controlled room, on a 12 h light-dark cycle with lights on at 6 a.m. to accommodate and recover from irradiation. At the age of 8 weeks mice were single-housed in Macrolon type III cages and 1 week later a wheel was introduced into each cage, as reported previously [10]. For home cage activity monitoring a separate group of 24 C57BL/6J mice was obtained from Charles River at the age of 8 weeks. Mice were single housed and given one week period of habituation, after which half of the cages were equipped either with a functioning or blocked wheel. Water and food were available *ad libitum*. Handling and testing of the mice were done during the light phase of the light-dark cycle. Full details of the study had been approved by the German animal welfare authorities (Regierungspräsidium Karlsruhe, approval number: AZ 35-9185.81/G144/04).

### Hippocampal irradiation

In preparation for the irradiation procedure, mice were anesthetized in groups of four at a time with a combination solution of Ketamine and Xylazine as described previously [48]. Radiation exposure was performed with a 6 MeV electron beam of a clinical linear accelerator (Siemens, Erlangen, Germany). Such therapeutic electron beams have radiobiological properties indistinguishable from high energy X-rays (i.e. 6 MV). But because of their much lower penetration depth in matter electrons proved to be more favorable in terms of animal shielding and, concomitantly, in generating a suitable dose profile over the hippocampal target volume by means of a small stereotactically defined opening (3.7 mm×11 mm slit) within the shielding plate (5 mm of lead). The slit was oriented perpendicular to the longitudinal axis of the animal. To keep the mice in a fixed position during exposure, anesthetized animals were placed in a special frame which allowed to reproducibly align the shielding plate (and the slit opening) with the animal physical geometry. Adjustment of the depth-dose profile was achieved by placing 2 mm of PMMA in the beam just above the shield plate openings and resulted in 100% nominal dose at the proximal and 75% at the distal border of the hippocampus (at +5 mm depth in the mouse), respectively. Dose fall-off to 50% was at +15 mm. The hippocampi of four mice could be irradiated simultaneously with one electron field of 10×10 cm^2^ beam area. The dose distribution beneath the opening was measured in a polystyrene phantom with a 0.016 cm^3^ ionization chamber (pinpoint chamber type 31014, PTW-Freiburg, Germany), with LiF thermoluminescence dosimetry (TLD 100H, Saint-Gobain Christals&Detectors, Solon, USA), and with radiochromic films (Gafchromic® HS14, International Speciality Products, Wayne (NJ), USA). A single dose of 10 Gy was administered at a dose-rate of 3 Gy/min (n = 36). Sham-treated mice were anesthetized and handled accordingly, only they received 0 Gy (n = 36).

### Behavioral testing

Five weeks after irradiation, mice were divided into runners (n = 36) and sedentary (n = 36) by giving them access to a running or a blocked wheel, respectively. Three weeks after voluntary running, mice were subjected to anxiety-related behavioral tests (open field, elevated O- maze, dark-light box).

### Open field test

Activity monitoring was conducted in a square shaped, white Openfield, measuring 50×50 cm^2^ and illuminated from above with about 50 Lux. Four Mice were tested simultaneously in four different Openfields. Groups were assigned randomly. The mice were placed individually into the middle of the arena and monitored by a Video camera (Sony CCD IRIS) for 16 min. The resulting data were analyzed using the image processing system EthoVision 3.0 (Noldus Information Technology, Wageningen, the Netherlands). For each sample, the system recorded position, object area, and the status of defined events. Parameters assessed for the present studies were frequency to center, total distance moved, velocity, and time spent in center.

### Elevated O-maze

The maze consisted of a gray plastic annular runway (width 6 cm, outer diameter 46 cm, 50 cm above ground level), covered with black cardboard paper to prevent mice from slipping off the maze. Two opposing sectors were protected by inner and outer walls of gray polyvinyl (height 10 cm). Animals were placed in one of the protected sectors and observed for 5 min. The maze was illuminated with 25 Lux. The latency for the first exit and total time spent in the open compartments were measured.

### Dark-light box

The Dark-light box consisted of two plastic chambers, connected by a small tunnel. The dark chamber measured 20×15 cm^2^ and was covered by a lid. The adjacent chamber, measuring 30×15 cm^2^, was white and illuminated from above with 600 Lux. Mice were placed into the dark compartment and latency to first exit, rearings/time in the lit (i.e. total number of rearings in the bright compartment divided through the time in this compartment), endexploration time (i.e. the latency until the mice reached the wall at the end of the bright compartment), and total time in the lit compartment were recorded for 5 min.

### Cat odor exposure for c-Fos induction

A plastic tube with cat feces was fixed in the center of the open field and mice were placed in the arena for 20 min and then returned to their home cages. Ninety minutes following exposure to cat odor, mice were deeply anaesthetized and perfused transcardially with 4% paraformaldehyde, and brains were processed for c-fos immunostaining as described below.

### Histological studies and quantification

Mice were anaesthetized by i.p. injection of ketamine and xylazine, and perfused transcardially as described previously [48]. Brains were removed, postfixed for ∼8 h in 4% paraformaldehyde, and kept in PBS overnight. Forty µm coronal sections were cut on a vibratome and kept at ­20°C in antifreeze solution until further processing. To evaluate neurogenesis, a primary rabbit polyclonal anti-Ki67-antibody (1∶5000; NCL-Ki67p, Novocastra, Newcastle upon Tyne, UK) and a primary goat polyclonal anti-DCX-antibody (Doublecortin; 1∶1000; sc-8066, Santa Cruz Biotechnology, Santa Cruz, CA, USA) were used. To demonstrate c-Fos staining, we used a primary rabbit polyclonal anti-c-Fos-antibody (1∶10.000; Oncogene Science, Cambridge, MA; AB-2). Every sixth section was processed free-floating, as described previously [48]. Sections stained for DCX were counterstained with hematoxylin solution (Fig. 2B). The quantitative analyses were performed as described previously [48]. Briefly, Ki67-immunoreactive cells along the subgranular zone of the dentate gyrus (Fig. 2A) were counted using a 100x oil-immersion objective. Cells in the uppermost focal plane of the section were excluded. Total cell number was calculated by multiplying the number of cells counted by the inverse of the section sampling fraction, i.e. 6. Quantification of c-Fos positive cells followed that of Ki67 quantification, and was performed in the dentate gyrus, CA3 and ventral CA1 throughout the hippocampus (Fig. 2C, 2D). Three animals were excluded from c-Fos counting due to poor tissue quality.

Total numbers of DCX-immunoreactive cells were estimated using the optical fractionator ([49]; StereoInvestigator 2000, Microbrightfield Inc., Williston, VT, USA) with a 100x oil-immersion objective. Counting frames (45×35 µm) were placed over the dentate gyrus at given intervals (135 µm along the x-axis and 105 µm along the y-axis). DCX-positive neurons, i.e. hematoxylin stained nuclei surrounded by a DCX-immunoreactive cytoplasm were counted throughout the section thickness but excluding cells in the uppermost focal plane (Fig. 2B).

### Determination of BDNF levels

After decapitation the hippocampus of both hemispheres was dissected and frozen on dry ice. BDNF protein levels were measured as described elsewhere [50]. Values were presented as means of both hemispheres.

### Home cage activity

Home cage locomotor activity was monitored using an infrared sensor (Mouse-E-Motion, Infra-E-Motion GmbH, Hamburg, Germany). Devices were places above the cages (30 cm from bottom) so that mice were detected at any position in the cage. Data were sampled every second. Following the observation, data were downloaded into a personal computer and further processed using Microsoft Excel. Locomotor activity was observed at the same time when the anxiety tests were performed in mice, i.e. between 10 a.m. and 6 p.m. in the light cycle (starting at 6 a.m. and ending at 6 p.m.). Illumination of the room was not changed.

### Statistical analysis

Statistical analysis was carried out using SPSS 16.0 (SPSS Inc., Chicago, IL). All data are reported as means ± S.E.M.. Differences between groups were detected using two-factorial analysis of variance followed by Bonferroni's or Fischer's LSD post hoc analysis, where appropriate or Students two-tailed t-test for comparing two groups. F- and p-values in the behavior section demonstrate the interaction effect of running and irradiation, showing that running-induced effects on behavior were absent in irradiated mice. Table S1 displays F- and p-values for all performed ANOVAs in the study. Significance was evaluated at a probability of 5% or less (<0.05).

## Supporting Information

Table S1 F- and p-values for all performed ANOVAs.
(0.06 MB DOC)
