# Supplementary material for: Deletion of Running-Induced Hippocampal Neurogenesis by Irradiation Prevents Development of an Anxious Phenotype in Mice
Source: PLoS One. 2010 Sep 16;5(9):e12769. doi: 10.1371/journal.pone.0012769 (PMC2940841; doi:10.1371/journal.pone.0012769)
Supplement: Table S1 — F- and p-values for all performed ANOVAs. (0.06 MB DOC) [file pone.0012769.s001.doc]

|  |  | Irradiation |  | Running |  | Interaction |  |
| --- | --- | --- | --- | --- | --- | --- | --- |
| Data |  | F | p | F | p | F | p |
| DCX |  | 63.904 | 0.001 | 16.093 | 0.001 | 9.315 | 0.006 |
| Ki67 |  | 79.385 | 0.001 | 0.574 | 0.454 | 0.000 | 0.987 |
| Open field | Frequency | 0.824 | 0.369 | 1.025 | 0.317 | 6.853 | 0.012 |
| Open field | Time in center 0-5 | 0.571 | 0.454 | 0.274 | 0.603 | 4.896 | 0.032 |
| Open field | Time in center total | 3.765 | 0.059 | 0.622 | 0.435 | 5.507 | 0.024 |
| Open field | Distance moved | 5.540 | 0.023 | 0.648 | 0.425 | 4.447 | 0.041 |
| Open field | Velocity 0-5 | 0.881 | 0.353 | 0.233 | 0.632 | 3.614 | 0.064 |
| Open field | Velocity 5-10 | 3.479 | 0.069 | 1.084 | 0.304 | 4.957 | 0.031 |
| Open field | Velocity 10-15 | 4.527 | 0.039 | 0.215 | 0.645 | 2.169 | 0.148 |
| O-maze | Latency first exit | 3.705 | 0.061 | 2.323 | 0.135 | 3.734 | 0.060 |
| O-maze | Time on open arms | 2.713 | 0.107 | 2.063 | 0.158 | 0.567 | 0.455 |
| DLB | Latency first exit | 0.049 | 0.826 | 6.202 | 0.017 | 6.471 | 0.015 |
| DLB | Time in lit | 0.102 | 0.751 | 7.662 | 0.008 | 9.317 | 0.004 |
| DLB | Endexploration | 0.061 | 0.806 | 15.470 | 0.001 | 11.427 | 0.002 |
| DLB | Rearings / time | 1.402 | 0.243 | 4.536 | 0.039 | 3.271 | 0.077 |
| c-Fos cage | DG | 0.065 | 0.803 | 84.896 | 0.001 | 0.083 | 0.778 |
| c-Fos cage | CA3 | 0.032 | 0.861 | 0.059 | 0.813 | 0.297 | 0.596 |
| c-Fos cage | CA1 | 0.086 | 0.775 | 3.850 | 0.073 | 0.998 | 0.338 |
| c-Fos odor | DG | 1.143 | 0.300 | 1.989 | 0.176 | 0.228 | 0.639 |
| c-Fos odor | CA3 | 0.729 | 0.405 | 11.359 | 0.004 | 2.205 | 0.156 |
| c-Fos odor | CA1 | 0.948 | 0.344 | 1.988 | 0.177 | 5.120 | 0.037 |
| BDNF |  | 0.135 | 0.717 | 4.906 | 0.039 | 0.013 | 0.910 |
